# Advance artificial time series forecasting model for oil production using neuro fuzzy-based slime mould algorithm

**DOI:** 10.1007/s13202-021-01405-w

**Published:** 2021-12-11

**Authors:** Ayman Mutahar AlRassas, Mohammed A. A. Al-qaness, Ahmed A. Ewees, Shaoran Ren, Renyuan Sun, Lin Pan, Mohamed Abd Elaziz

**Affiliations:** 1 School of Petroleum Engineering, China University of Petroleum (East China), Qingdao, China; 2 State Key Laboratory for Information Engineering in Surveying, Mapping and Remote Sensing, Wuhan University, Wuhan, 430079 China; 3 Department of Computer, Damietta University, Damietta, Egypt; 4 Faculty of Earth Resources, China University of Geosciences, Wuhan, China; 5 Department of Mathematics, Faculty of Science, Zagazig University, Zagazig, 44519 Egypt; 6 Artificial Intelligence Research Center (AIRC), Ajman University, Ajman, 346 United Arab Emirates; 7 Department of Artificial Intelligence Science & Engineering, Galala University, Suze, 435611 Egypt; 8 School of Computer Science and Robotics, Tomsk Polytechnic University, Tomsk, 634050 Russia

**Keywords:** ANFIS, Slime mould algorithm, Oilfield, Time series forecasting, Oil production

## Abstract

Oil production forecasting is an important task to manage petroleum reservoirs operations. In this study, a developed time series forecasting model is proposed for oil production using a new improved version of the adaptive neuro-fuzzy inference system (ANFIS). This model is improved by using an optimization algorithm, the slime mould algorithm (SMA). The SMA is a new algorithm that is applied for solving different optimization tasks. However, its search mechanism suffers from some limitations, for example, trapping at local optima. Thus, we modify the SMA using an intelligence search technique called opposition-based learning (OLB). The developed model, ANFIS-SMAOLB, is evaluated with different real-world oil production data collected from two oilfields in two different countries, Masila oilfield (Yemen) and Tahe oilfield (China). Furthermore, the evaluation of this model is considered with extensive comparisons to several methods, using several evaluation measures. The outcomes assessed the high ability of the developed ANFIS-SMAOLB as an efficient time series forecasting model that showed significant performance.

## Introduction

Forecasting oil production is a significant step for controlling the management of the cost-effect and monitoring the operation of petroleum reservoirs. Consequently, the forecasting of oil production facilitates the reservoir engineers to design plausible projects, which triggers to prevent the blind investment and attains sustainable evolution. Therefore, accurate forecasting of a petroleum reservoir is highly required to control and manage the effective cost of the oil reservoirs. The reservoir properties, including porosity, permeability, compressibility, fluid saturation, and other well operational parameters have a significant effect on oil production. Therefore, it is challenging to forecast future oil production accurately because of the reservoir’s complexity, and uncertain subsurface conditions (Liu et al. 2020). Numerical reservoir simulation (NRS) and decline curve analysis (DCA) are conventional methods and are commonly used to predict oil production (Doublet et al. 1994; Cumming 2013; Cancelliere et al. 2011). However, both conventional methods still have some limitations,that affect the accuracy of the forecasting performance. Thus, the effective development of oilfields requires an accurate development approach to predict the oil production precisely which assists to select the proper oil recovery methods to increase oil production, and enhance oil transfer from subsurface to surface. Also, it leads to extending the oilfield’s life cycle and energizing the economy profit. The (DCA) method utilizes the empirical equations to fit the oil production historical data to characterize the whole reservoir’s production mechanism (Tomomi et al. 2000). Moreover, matching the historical production data of the oil wells is a significant challenge, and consuming time, even if the history well’s production presents perfect matching. Nevertheless, the potential of calculating the uncertain predictions is possible, even if there are complex and unstable production conditions (Li et al. 2003). On the other hand, the accuracy of (NRS) is robust and reliable to predict oil production; however, accuracy and reliability depend on the static geological model and the quality of dynamic reservoir simulation models, because the development construction of the static geological models is extremely difficult (Hutahaean et al. 2015, 2016; Al Rassas et al. 2020). Furthermore, the parameterization approaches of the static geological model, and the combing means of objective components have a significant effect on the reservoir history matching, and reservoir predicting (Liu et al. 2020; Song et al. 2020; Kalra et al. 2018). Although multi-objective optimization issues can be addressed effectively, a perfect reservoir history matching model can trigger to cause a bad prediction. The process of history matching is a challenge and required too much time to deal with extensive work.

Deep learning approaches and their implementation have recently grown in the petroleum industry, particularly in reservoir engineering applications (Alkinani et al. 2019), including predicting porosity and permeability (Erofeev et al. 2019; Ahmadi and Chen 2019), Pressure-Volume, Temperature (PVT) (Goda et al. 2003; Alkinani et al. 2019), sensitive analysis and history matching, and forecasting oil production (Ahmadi and Bahadori 2015; Montgomery and O’sullivan 2017; Guo et al. 2018).

Furthermore, the powerful development of deep learning, with the significant evolution of the deep learning algorithms, was introduced to the petroleum industry to overtake the complication issues of traditional methods (Song et al. 2020). Additionally, in literature, various machine learning and deep learning methods had been presented for forecasting oil production (Liu et al. 2020; Wang et al. 2020; Sagheer and Kotb 2019; Wang and Chen 2019). Song et al. (2020) employed Long Short-Term Memory (LSTM) for forecasting oil production time series. In (Alalimi et al. 2021), a modified Random Vector Functional Link network was proposed for time series prediction. This model was applied for oil production in Tahe oilfield, China. Liu et al. (2020) used LSTM with Empirical Mode Decomposition Ensemble to forecast oil production. In (Cc et al. 2013), an oil production forecasting model, namely, the higher-order neural network was proposed. Masini et al. (2020) proposed a combination of algorithms including clustering and density-based clustering with Artificial Intelligence techniques including (Long Short-term memory cells algorithm (LSTM), Vertical Flow Performance (VFP)) to demonstrate assisted production forecasting from the real-time data. McKenna et al. (2020) employed three different levels of uncertainty, including (facies geometry, permeability distribution, and reservoir rock heterogeneity) to assess their influence on reservoir evaluation and prediction. Sequential Gaussian simulation and Kriging probability- field were used to estimate and demonstrate previous uncertainty levels. Fan et al. (2021) presented a hybrid model which considered the benefits of linearity and non-linearity and the effect of manual operations by incorporating the ARIMA (autoregressive integrated moving average) and the LSTM. Moreover, four evaluation methods were utilized to compute the forecasting accuracy.

Rădulescu et al. (2020) proposed an econometric approach for forecasting oil production to permit decision-makers and oil product stakeholders to take liability for the production in OECD partner countries. This liability is perceived from various perspectives: political, economic, environmental, military, social, etc. Sagheer and Kotb (2019) proposed deep LSTM to address the drawbacks of conventional prediction techniques and present accurate predictions. Semenychev et al. (2017) Elucidated the complexities of modeling and forecasting the petroleum industry by integrating several production trend models and models of fluctuation. These methods increase the production forecasting accuracy by incorporating the fluctuation components models and controlling the model’s evolution and fluctuation. Allen (2020) proposed a data-driven approach as an alternative to traditional production prediction methods. They presented a proxy-well model to predict the production by choosing significant parameters and reservoir data as independent predictor variables. After that, principal component analysis (PCA) was employed to obtain the relevant features,and was employed to estimate the cumulative productions. Wang et al. (2018) a hybridization model of a nonlinear and linear prediction approach was proposed to establish predicting techniques in two-stages, integrating nonlinear grey approach accompanied by mentalism idea to establish nonlinear metabolism grey approach and incorporating it with ARIMA. Al-Shabandar et al. (2021) presented a new model for prediction oil production using a deep-gated RNN that comprises several hidden layers, in which each one has a set of nodes. This model had been evaluated with long-term time-series data.

Negash and Yaw (2020) proposed a new model for oil production forecasting employing artificial neural networks (NNs), which require a physics-based feature extraction to predict fluid production and to boost the forecasting effect. Additionally, there are also other models, such as (Suhag et al. 2017; Liu et al. 2020; Karasu et al. 2020; Male 2019; Aizenberg et al. 2014).

Furthermore, the application of DL in the petroleum industry was not only apply for forecasting oil production, however, recently different DL methods were employed to simulate the carbon emission and reduction (Wang et al. 2022, 2020, 2021), as well as the impact of energy consumption during the COVID-19 pandemic (Wang et al. 2021)

In this study, we develop a time-series forecasting approach using an improved ANFIS (adaptive neuro-fuzzy inference system) (Jang 1993) for oil production. We utilize an enhanced version of the lately proposed metaheuristic optimization method, Slime mould algorithm (SMA) based on the opposition-based learning (OBL).

In recent years, the ANFIS model has been adopted in various forecasting applications, such as, oil consumption (Al-Qaness et al. 2018; Al-qaness et al. 2019), COVID-19 cases (Al-Qaness et al. 2020), influenza cases (Al-qaness et al. 2020), and others (Zhou et al. 2019). The SMA is a recently developed optimization algorithm, presented by (Li et al. 2020). It simulates the behavior of initializing negative and positive feedback of the slime mould propagation waves of slime mould depending on bio-oscillator to form optimal paths to connect foods using efficient exploitation ability and valued exploratory propensity. Due to its competitive performance in solving complex optimization problems, it has been adopted in different applications.

The modified ANFIS is improved using an enhanced version of the SMA using the OLB; thus, it is named SMAOLB-ANFIS. It works by initializing a set of solutions; each solution represents the configuration from ANFIS parameters. We evaluate each solution using 70% of the samples as a training set. The solution that has the smallest fitness value is considered the best solution. Thereafter, the OLB operators are employed to boost the current population, and then SMA operators are used to improve current solutions till meeting terminal conditions. The best ANFIS configuration ( the best solution) is estimated using 30% of the samples as a testing set. The data used in this study are real-datasets for Masila oilfields in Yemen, and Tahe oilfields in China, provided by local partners. The proposed forecasting approach achieved significant performance using several evaluation metrics with comparisons to other methods.

The main contribution of the current study is:

- Present an efficient forecasting model for oil production based on a new improved ANFIS model.
- Propose an enhanced SMA algorithm to optimize ANFIS parameters using the OBL intelligence search technique.
- We evaluate the proposed forecasting model with two real-world datasets from two different oilfields in Yemen and China. Also, we compare the SMAOLB to several optimization methods to verify its performance.

## Backgrounds

In this section, we give a brief description to the applied methods, as follows.

### ANFIS

The ANFIS approach was established by Jang (1993) as a new artificial network (ANN). The ANFIS model’s structure is considered incorporation of ANN and Fuzzy Inference Systems (FIS). Furthermore, “IF-THEN rules” are applied to generate a mapping for inputs and outputs, identified as the “Takagi–Sugeno inference model”. This renders to substantiate that the ANFIS approach is more convenient and reliable to process data as it has a robust learning capability. As stated by these characteristics, the ANFIS approach has been implemented in many applications.

**Fig. 1:**
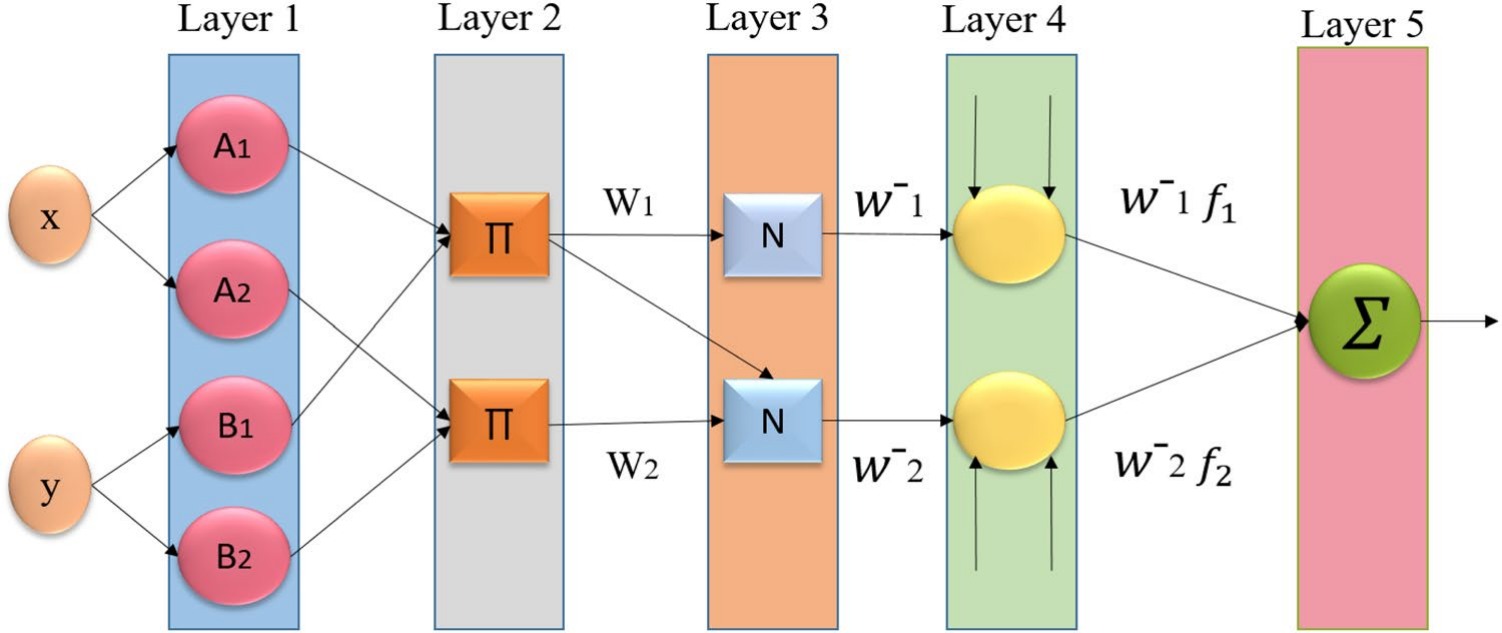
The basic ANFIS structure

In the common ANFIS workflow, as drawn in Fig. 1, the Layer 1 input is represented by *x* and *y*, where $L_{1i}$ indicates the outputs of *i* node. The ANFIS mathematical model is expressed as follows:

1 $$\begin{aligned} L_{1i}= & {} \mu _{A_i} (x), \, i= 1,2 ,\; L_{1i}=\mu _{B_{i-2}} (y), \, i= 3,4 \end{aligned}$$

2 $$\begin{aligned} \mu (x)= & {} e^{-(\frac{x-\rho _i}{\alpha _i} )^2}; \end{aligned}$$

where $\mu$ indicates the generalized Gaussian membership function. The membership values of $\mu$ are defined by $A_i$ and $B_i$, and $\alpha _i$ and $\rho _i$ refer to premise parameter set.

More so, Eq. (3) can be utilized for the second layer:

3 $$\begin{aligned} L_{2i}=\mu _{A_i} (x) \times \mu _{B_{i-2}} (y) \end{aligned}$$

The output of the third Layer is calculated as :

4 $$\begin{aligned} L_{3i}= \overline{w}_i = \frac{\omega _i}{\sum _{(i=1)}^2 \omega _i} , \end{aligned}$$

In which $w_i$ represents *i*th output from the layer 2.

Furthermore, the output of layer is generated by Eq.5.

5 $$\begin{aligned} L_{4,i}=\overline{w}_i f_i=\overline{w}_i(p_i x+q_iy+r_i) \end{aligned}$$

In which *f* indicates a function which use input and parameters of the network as inputs. $r_i$, $p_i$, and $q_i$ indicate *i* consequent parameters.

Finally, layer 5 generates the output that is computed as in Eq. (6).

6 $$\begin{aligned} L_5=\sum _i \overline{w}_i f_i \end{aligned}$$

### Slime mould algorithm

In 2020, SMA was proposed by (Li et al. 2020) as an alternative natural-inspired optimization technique that can be implemented to solve different optimization issues. It mimics the performances of slime mould’s Oscillation and their propagation wave feedback depending on the bio-oscillator, and generates the optimum routes to connect food. It has three primary phases:

1. Phase 1(Approach food): This phase can be presented as in Eq. 7, to define approaching behavior of slime mould.
  7 $$\begin{aligned} Z_{t+1}=\left\{ \begin{matrix} Z_{b}(t)+v_{b}.\left( W.Z_{A}(t)-Z_{B}(t) \right) &{} r<p \\ v_{c}.Z_{t} &{} r\ge p \end{matrix}\right. \end{aligned}$$
  in which $v_{b}\in [-a,a]$ represents random value, $v_{c}$ indicates *t* random value that is reduced from 1 to 0, and *t* is the current iteration number. Moreover, $Z_{b}$ represents the best solution. The solutions of the Slime are indicated by *X*. $Z_{A}$ and $Z_{B}$ are two random selected solutions. Additionally, *W* represents the slime mould weight. Whereas *p* is calculated using Eq. 8:
  8 $$\begin{aligned} p= \tanh \left| S(i)-DF| \right. , \, i=1,2,...,n \end{aligned}$$
  in which *S*(*i*) indicates the fitness value of *i*-th solution, and *DF* is the best fitness value.
  The $v_{b}$ is computed using Eq.9:
  9 $$\begin{aligned} v_{b}= & {} [-a,a] \end{aligned}$$
  10 $$\begin{aligned} a= & {} arctanh \left( -\left( \frac{t}{max_t} \right) +1 \right) \end{aligned}$$
  *W* is computed as follows:
  11 $$\begin{aligned} W(SmellIndex(i))= & {} \left\{ \begin{matrix} 1+r \log ((b_F-S(i))/(b_F-w_F)+1) &{} condition \\ 1-r \log ((b_F-S(i))/(b_F-w_F)+1) &{} others \end{matrix} \right. \end{aligned}$$
  12 $$\begin{aligned} SmellIndex= & {} sort(S) \end{aligned}$$
  here, *condition* indicates that *S*(*i*) is ranked in first half of *X*, where *r* is randomly generated in [0,1]. More so, $b_F$ indicates the best local fitness value, and $w_F$ is the worst local fitness value. *SmellIndex* stores the sorted fitness value.
2. Phase 2 (Wrap food): This phase is emplyed to simulates the updating position process of the slime mould. It can be represented as in Eq.13:
  13 $$\begin{aligned} Z^{*}=\left\{ \begin{matrix} rand (UB-LB)+LB &{} rand<z \\ Z_b (t)+v_b(WZ_A(t)-Z_B (t)) &{} r<p\\ v_c Z(t) &{} r\ge p \end{matrix}\right. \end{aligned}$$
  in which *LB* and *UB* indicate the limits of search space, whereas rand and $r\in [0, 1]$ can be randomly generated.
3. Phase 3 (Oscillation): during this stage, the $v_b$ is oscillating in $[-a,a]$, whereas $v_c$ is oscillating in $[-1, 1]$.
  Algorithm 1 presented the entire steps of the SMA.

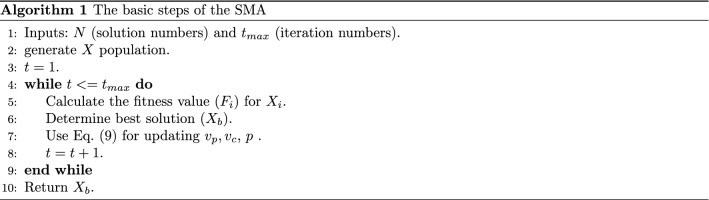

### Opposition-based learning

The OBL (Tizhoosh 2005) is an artificial intelligence technique that can be utilized to improve various methods of optimization (Ewees et al. 2018). The OBL strategy is based on the current approach to creating new opposition solutions for the given issue. This approach aims to select the optimal candidate solution by achieving the optimum fitness score to obtain the ideal solution (Abd Elaziz et al. 2017). The *X* opposite value for the real value, where *X* $\in$ [*UB*,*LB*], is computed as shown in Eq. (14).

14 $$\begin{aligned} X=UB+LB-X \end{aligned}$$

Opposite point: Suppose *X* = ($x-1$, $x-2$,..., $x-n$) is a multi-dimensional space point, in which $x-1$, $x-2$,..., $x-Dim$ $\in$ *R* and $x-j$ [$UB-j$,$LB-j$], *j* $\in$ 1, 2,..., *Dim*. Thus, This formulation is utilized by adding Equation (15) to resolve n-dimensions.

15 $$\begin{aligned} \overrightarrow{x_j}=UB_j+LB_j-x_j, \quad \quad where \quad j=1....D. \end{aligned}$$

Furthermore, two solutions are given (*x* and $x_{old}$) and compared in the optimization process based on their fitness functions. Then the best solution is saved, whereas other solutions are removed. If *f*(*x*) $\ge$ *f*($x_{old}$) is stored for maximization, then *x* is stored; otherwise, $x_{old}$ is stored.

### Proposed SMAOLB-ANFIS model

**Fig. 2:**
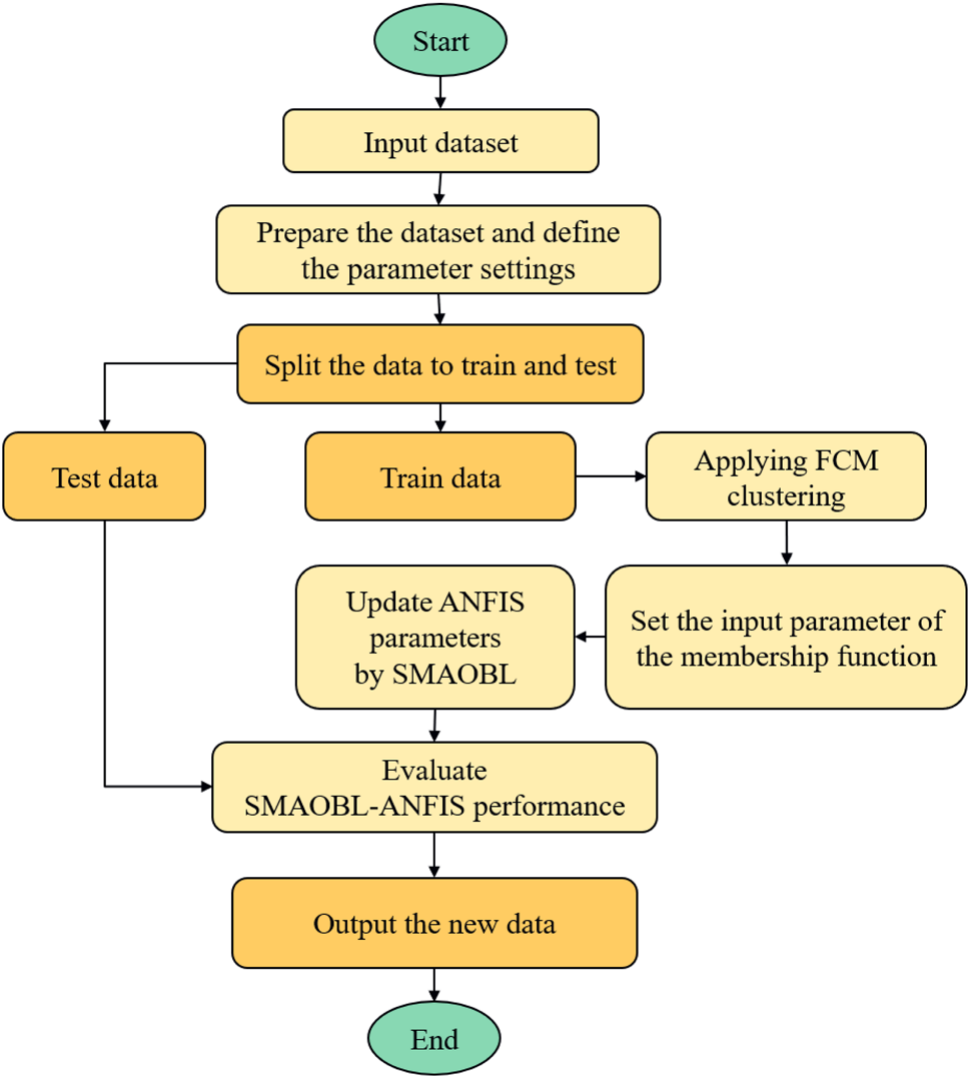
The steps of the SMAOBL-ANFIS

The developed forecasting oil production model is discussed in this section. The proposed model depends on improving the performance of ANFIS based on enhanced SMA according to the value OBL. The main target of using SMAOBL is to the parameters of ANFIS as in Fig. 2.

The first step in the developed model, named SMAOLB-ANFIS, is to split the oil production dataset into training and testing sets, then using the training set during the learning stage. In this stage, the developed SMAOLB-ANFIS constructs a population *X*, which has a set of *N* solutions; each of one refers to one configuration from the parameters of ANFIS. The next step is to assess the performance of constructed ANFIS according to the current configuration $X_i$ by using the following fitness function.

16 $$\begin{aligned} MSE = \frac{1}{N_a} \sum _{i=1}^{N_s}(T_i-P_{i})^{2} \end{aligned}$$

where *T* and *P* denote the targets and predicted outputs, respectively. $N_a$ indicates the total number of samples of the training set.

The next process is to update the current population *X* by applying the modified SMAOBL. This is achieved by using the operators of SMA as discussed in Algorithm 1. Followed by applying the OBL operator as discussed in Eq. (15). Because OBL needs more computational time, so the developed SMAOBL uses OBL only during the exploration phase. The next step is to check the terminal condition and if it is not satisfied, then repeat the updating steps; Otherwise, return the best configuration which represents $X_b$. Thereafter, apply the testing set to the best configuration $X_b$ and evaluate its quality by predicting the oil production. The description of the developed ANFIS is presented in Algorithm 2. 
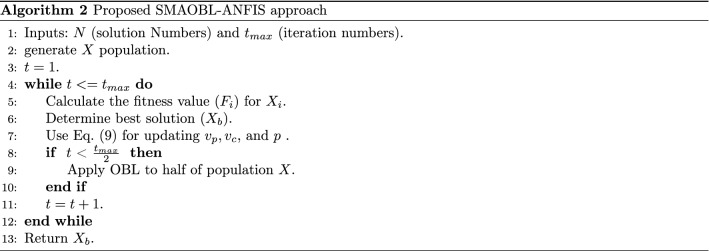

## Evaluation experiments

### First study area

**Fig. 3:**
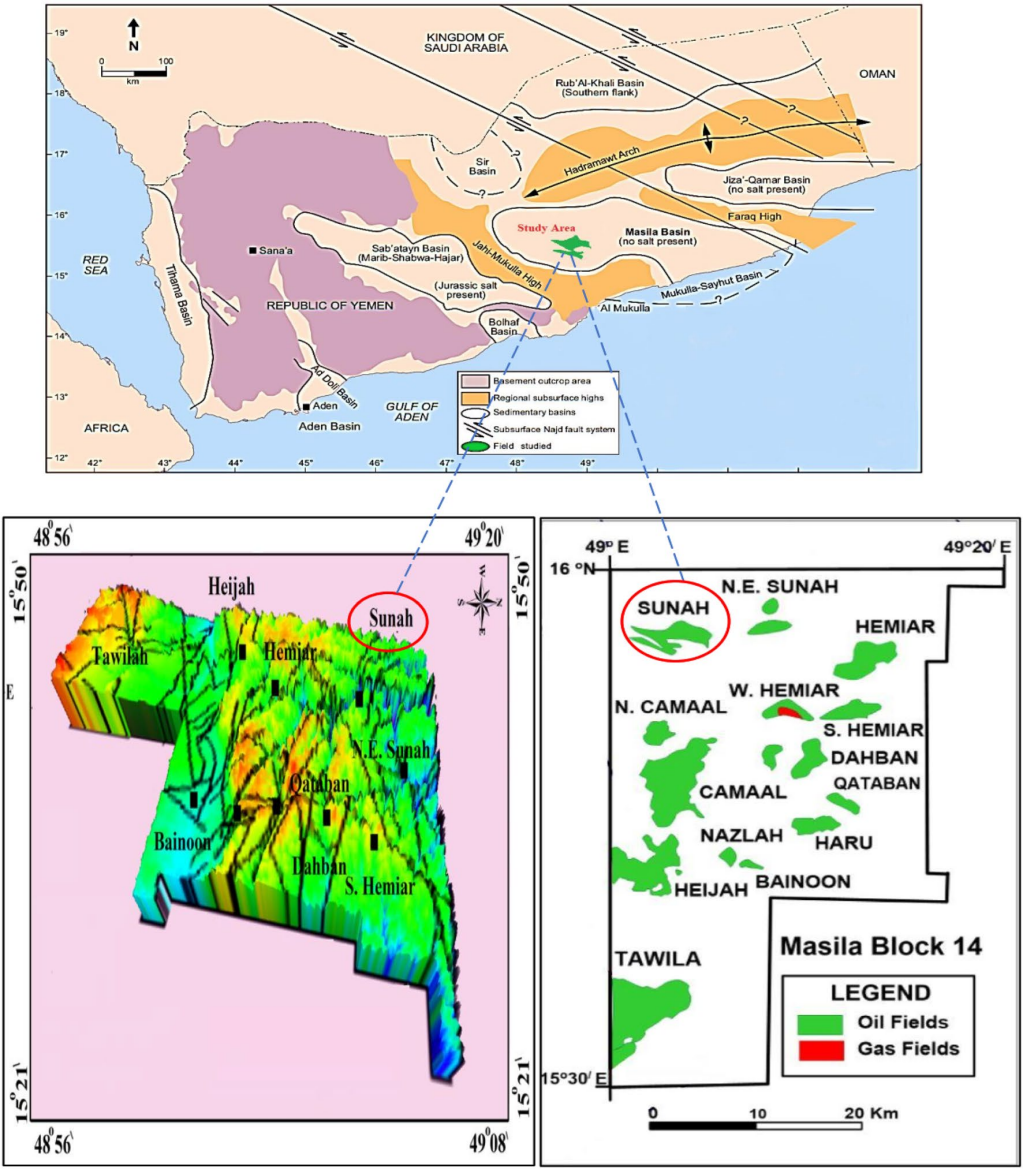
First Study area, Masila Basin oilfield, Yemen

The first case study or study area is the Masila Basin, Yemen. It is one of the onshore basins located in Hadrammot governorate. It occupied about 1250 km$^2$ , and it can be considered as one of the Mesozoic sedimentary basins. It was generated as a rift-basin associated with the Mesozoic breakup of Gondwanaland and its development in the Indian Ocean throughout the Jurassic and Cretaceous. The Mesozoic and Cenozoic sequence in Yemen sedimentary basins are widely exposed. Many researchers have studied the lithostratigraphic structure in the Masila Basin includes Sunah oilfield (Hakimi et al. 2014, 2017; Al-Areeq and Maky 2015). Block 14 in the Masila basin comprised 20 producing fields, as illustrated in Figure 3. The Sunah oilfield is located in the northwest portion of the Masila block. The S1A formation is made up of shelf sands with tidal and longshore impacts that range in thickness from 25 to 40 feet. Figure 3 presents the study area of the Masila basin - Block 14, Sunah oilfield.

#### Geological setting

**Fig. 4:**
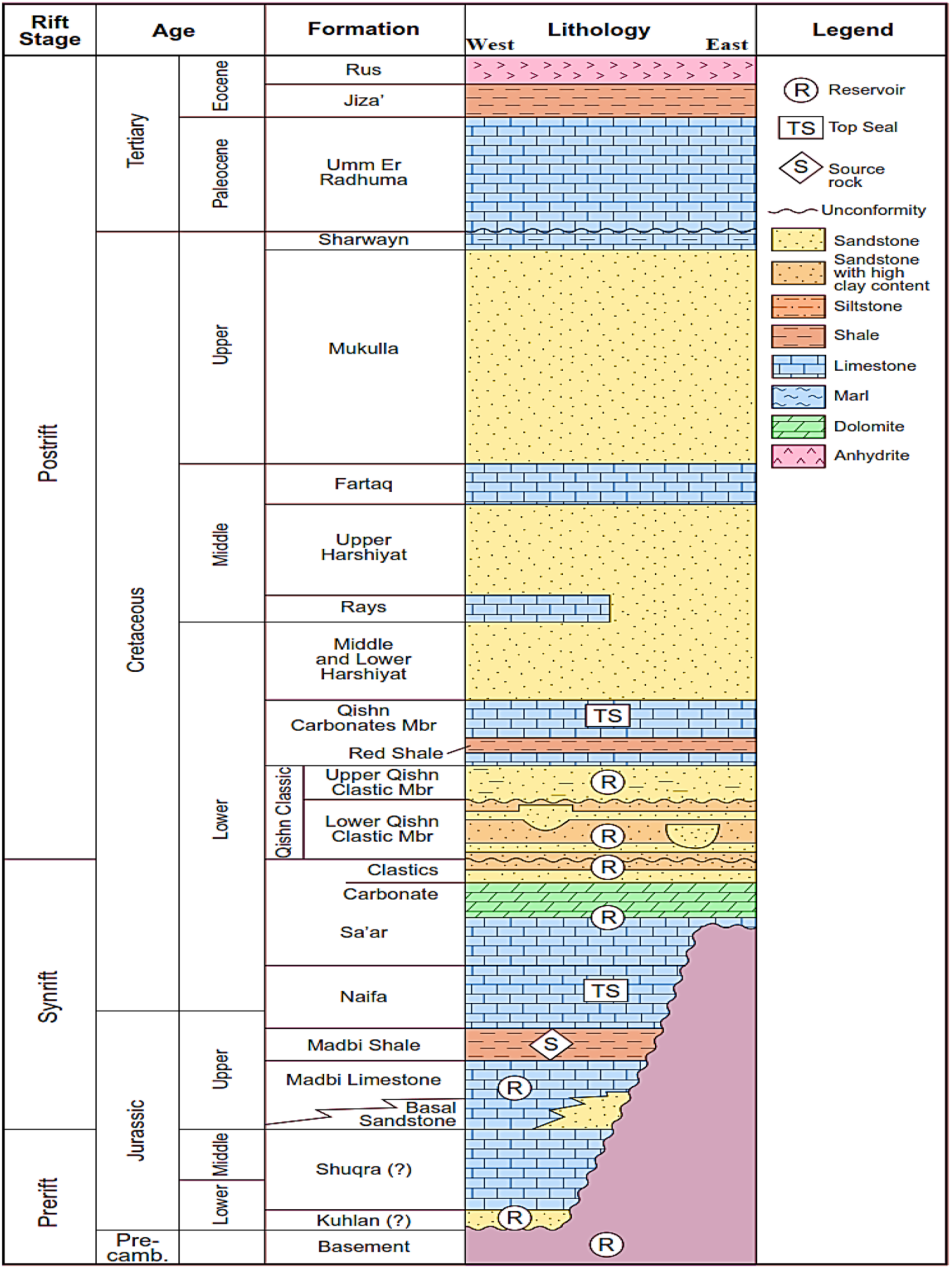
Geological setting of the first study area

The geological characteristic of Masila oilfield has a substantial role in determining the hydrocarbon zones throughout Masila oilfield. The hydrocarbon occurrence and movement were mainly monitoring by several attributes, including petrophysical properties, facies, faults, folding, and fractures. The Masila block is located in Hadhramaut city and ranks as the most active oilfields (Figure 4) (Hakimi et al. 2011).. The lithostratigraphic unit is varied in the era from Proterozoic to Tertiary. It is divided into different mega sequences, such as pre-rift, post-rift, and syn-rift. The S1A formation (Madbi Formation) is formed in the Upper Jurassic. Structurally, the Masila block was influenced by several fault trends northwest and southeast as a consequence of the red sea and Aden’s Gulf rifting throughout the tertiary time Masila basin. More so, the Jurassic and Lower Cretaceous strata reflect post-Pangaea separated in Yemen’s southern part, particularly in the Masila block. The block development was generated by cracking during the Early Cretaceous and Late Jurassic. Yemen was encountered rifting twice in the Mesozoic and three times in the Tertiary time. The Mesozoic rifting basin trends from west to east, Sayun- Masila, and Jiza Qamar basin.

### Second study area

**Fig. 5:**
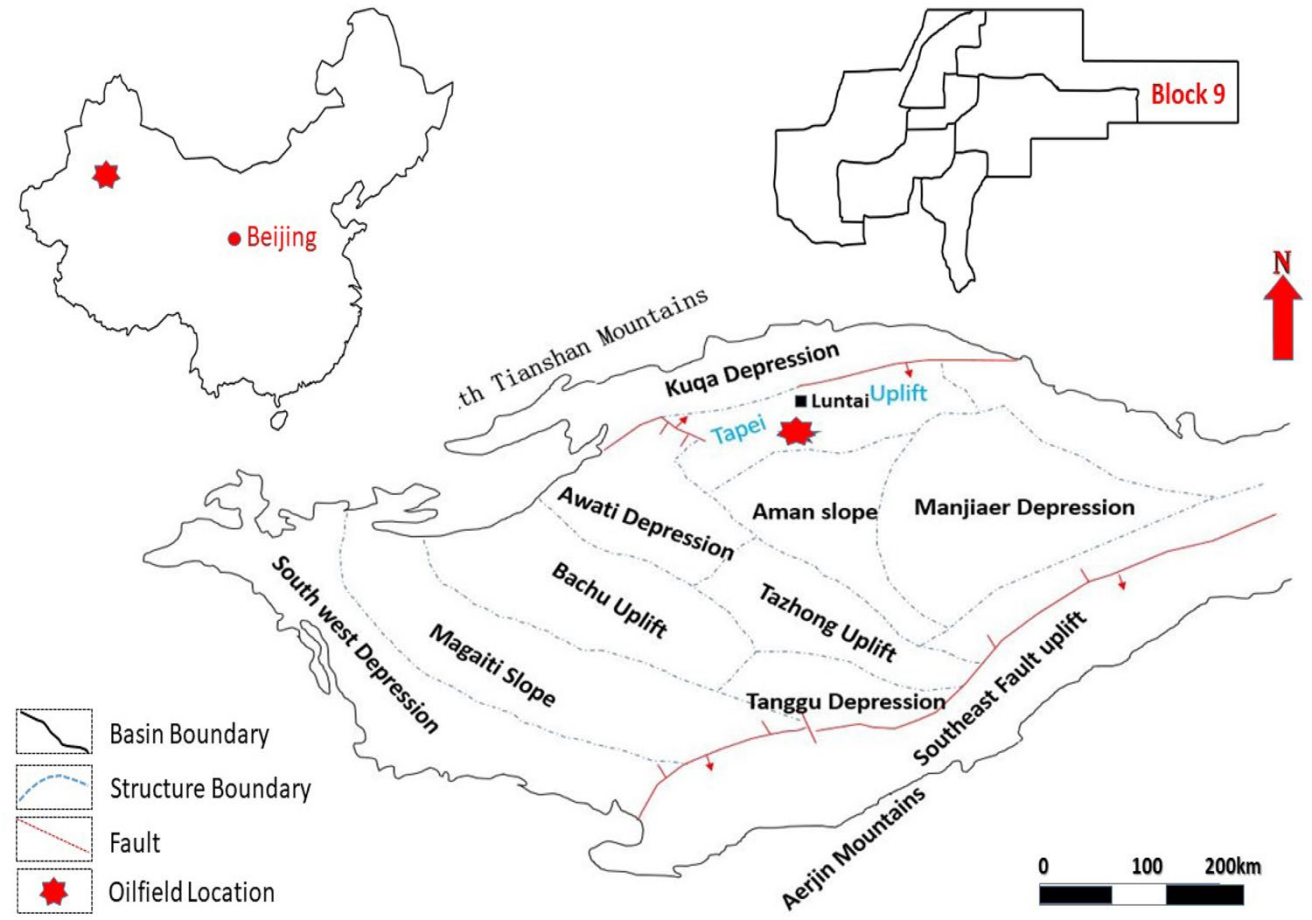
Second Study area (Tahe oilfield, Block 9, China)

Taha oilfield was discovered in 1990s with total proven reserves of approximately $600\times 10^6$ tones. Taha oilfield is situated in Luntai County, Xinxiang province (Höök et al. 2010; Tian et al. 2017). Triassic Oil Formation in the Block-9 of Tahe Oilfield is located about 60 kilometers(km) away from the Luntai country, and its eastern longitude lies between $84^\circ ~13'~9`` -84^\circ ~18'~52''$ and northing latitude $41^{\circ }~15'~56``-41^{\circ }~16'~4''$. Triassic reservoir block-9 was discovered in 2002. Triassic reservoir, block-9 is a sandstone reservoir, which is considered a favorable place for Hydrocarbon accumulations. The oil production was started in 2002, divided into four stages of development, including the pre-production phase, upper-middle-class, stable production phase, and regressive phase (Li and Pan 2017; Yu et al. 2017). Figure 5 shows the location of this oilfield.

#### Geological setting

Geologically, block 9 in the Taha oilfield is a sandstone reservoir that belongs to the Triassic era. Block 9 consists of 10 normal faults, three large normal faults are extended from the north to the east direction, and the others are secondary normal faults. The reservoir traps are anticline and are asymmetrical on both sides. The sedimentary lithofacies is composed of four types of lithological units including channel sand, levee sand, channel margin, and clay. Block 9 has good petrophysical properties including porosity and permeability with an average proportion of 16.95% and 330.25 mD respectively. The detailed description of the geological setting of this oilfield was described by (Lin et al. 2012).

### Evaluation metrics

To validate the ability of the developed method to predict the oil production, a set of performance metrics is employed. These measures are the Standard deviation (std), Mean Square Error, Mean Absolute Percentage Error, Mean Absolute Error, and Coefficient of Determination. and their formulations are given in Table 1.

**Table 1:** Performance Metrics

Performance measure	Formula
Mean Square Error (MSE)	$MSE = \frac{1}{N}\sum _{i=1}^{N_s}(Py_i-Y_{i})^{2}$
Mean Absolute Error (MAE)	$MAE = {\frac{1}{N} \sum _{i=1}^{N_s} \vert Py_i - Y_{i} \vert }$
Mean Absolute Percentage Error (MAPE)	$MAPE = {\frac{1}{N} \sum _{i=1}^{N} \vert \frac{ Py_i - Y_{i} }{YP_i} \vert }$
Coefficient of Determination ($R^2$)	$R^{2} = 1 - \frac{\sum _{i=1}^{n}(Y_{i}-Py_{i})^{2}}{\sum _{i=1}^{n}(Y_{i}-\overline{Y}_{i})^{2}}$
Standard deviation (Std)	$Std = \sqrt{\frac{1}{{{N}}}\sum \limits _{k=1}^{{N}} {{{( {Y_k - \overline{Y}} )}^2}}}$

### Results

The experiment results are calculated based on four real datasets to forecast oil products for Yemen and China (one dataset for Yemen and three for China). The Yemen dataset consists 341 records collected yearly between 1993 - 2015, whereas the China datasets, namely TK905H, TK906H, and TK907H, contain 4108, 4143, and 3838 records, respectively collected daily from 2003 to 2014. The averages of each dataset are as following: Yemen = 31946.95, TK905H = 29.06, TK906H = 33.53, and TK907H = 38.04. These data vectors are formatted to be used in time-series forecasting by applying the auto-correlation function (ACF). Therefore, 7-lags are applied in preparing the China data to be used in the forecasting process whereas, 2-lags are applied for Yemen data. In addition, the dataset is divided into training and testing sets using 10-cross-validation.

#### Yemen oil field

To evaluate the proposed SMAOLB-ANFIS as a time series forecasting model, we used real datasets collected from Masila oilfields, Yemen. Additionally, we compared the SMAOLB to other models, including the traditional ANFIS, and several ANFIS improved versions using several optimization techniques, namely, SMA, genetic algorithm(GA), particle swarm optimization algorithm (PSO), and whale optimization algorithm (GWO), and sine cosine algorithm (SCA).

Table 2 shows the evaluation results of all compared algorithms in terms of RMSE, MAE, MAPE, $R^2$, STD, and computational time (CPU time). For RMSE, the proposed SMAOLB obtained the best results, followed by PSO, GA, SMA, GWO, ANFIS, and SCA, respectively. It is clear that SMAOLB outperforms the traditional SMA and traditional ANFIS, which confirmed the advancements of the proposed method, which is improved by using the operators of the OLB. In case of MAE, The proposed SMAOLB also achieved the best results, followed by PSO, GA, SMA, ANFIS, GWO, SCA, respectively. For $R^2$, it is clear that the proposed SMAOLB achieved the best results with 99.6%. The PSO obtained the second rank, where PSO and GA obtained the third rank. The ANFIS and GWO obtained the fourth rank, and finally, SCA came in the last rank. For *STD*, SMAOLB also obtained the best rank, followed by PSO, GA, ANFIS, SMA, GWO, and SCA, respectively. In contrast to previous records, for computational time, GWO obtained the shortest time, followed by SCA, PSO, GA, SMA, and SMAOLB. This is because the applications of OLB enhanced the search process of the SMA to obtain optimal solutions Table .

**Table 2:** Results of Yemen Oil fields

**Alg.**	RMSE	MAE	MAPE	$R^2$	Std	Time
SMAOLB	**18.2429**	**15.773**	**0.03754**	**0.99600**	**0.062**	5.225
ANFIS	30.9510	27.698	0.06574	0.99558	2.365	-
SMA	24.8025	21.510	0.05115	0.99540	9.285	5.173
PSO	18.3333	15.778	0.03755	0.99517	0.079	2.872
GA	18.3410	15.785	0.03757	0.99517	0.145	3.152
SCA	174.1140	172.580	0.40997	0.99535	113.315	2.848
GWO	30.6688	26.820	0.06375	0.99540	14.751	2.778

**Fig. 6:**
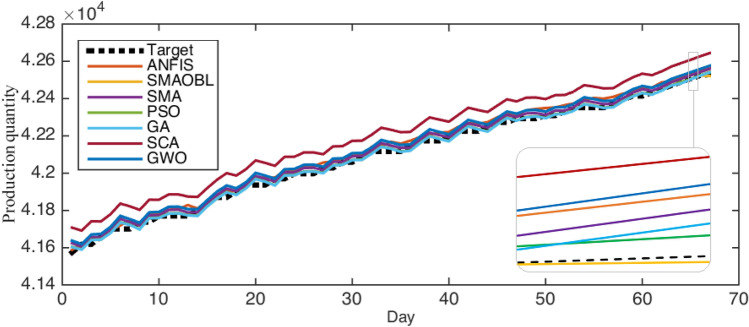
Results of the SMAOLB-ANFIS and the compared model

Additionally, Figures 6 illustrates the forecasting results of the SMAOLB-ANFIS and the compared models. As shown from this figure, the proposed SMAOLB obtained the nearest values of the target (real value).

#### Tahe oil field, China

For further evaluation of our proposed model, we use another data for three wells in the Tahe oilfield, China. Tables 3-5 show the results of all algorithms for Tahe oilfield, China. As illustrated in Table 3, for the well TK905H, the proposed SMAOLB obtained the best RMSE value. Then, the PSO came in the second rank, where the GA obtained the third rank. More so, the traditional SMA obtained the fourth rank, where the SCA and traditional ANFIS recorded the fifth and sixth ranks, respectively. For the TK906H and TK907H wells, SMAOLB also came in the first rank, followed by PSO, GA, SMA, ANFIS, and SCA. From Table 4, for TK905H and TK906, we see that the SMAOLB achieved the best MAE values, followed by PSO, GA, SMA, SCA, and ANFIS. For TK907, SMAOLB is also the best, followed by PSO, GA, SMA, ANFIS, and SCA. Furthermore, Table 5 indicates that the developed SMAOLB obtained the best $R^2$ value for the three wells.

**Table 3:** RMSE of three oil wells in Tahe oil Field, China

RMSE	ANFIS	SMA	SMAOBL	PSO	GA	SCA
TK905H	3.28086	2.49788	**2.31342**	2.31673	2.31725	2.63118
TK906H	1.84751	1.13141	**1.12591**	1.12736	1.12754	1.89347
TK907H	1.82949	1.76135	**1.74782**	1.75519	1.76201	2.16795

**Table 4:** MAE of three oil wells in Tahe oil Field, China

MAE	ANFIS	SMA	SMAOBL	PSO	GA	SCA
TK905H	2.01827	1.27891	**1.13691**	1.13795	1.14554	1.45180
TK906H	1.13836	0.70205	**0.69289**	0.69851	0.70003	1.32258
TK907H	0.89436	0.80083	**0.75259**	0.78672	0.79756	1.19983

**Table 5:** $R^2$ of three oil wells in Tahe oil Field, China

$R^2$	ANFIS	SMA	SMAOBL	PSO	GA	SCA
TK905H	0.85207	0.88392	**0.89842**	0.89776	0.89794	0.88185
TK906H	0.96028	0.98083	**0.98094**	0.98092	0.98090	0.96538
TK907H	0.91711	0.92205	**0.92225**	0.92182	0.92166	0.90438

### Statistical tests

For further analysis, in this section, the Friedman test is employed to test the robustness of the SMAOLB and other compared algorithms depending on all applied evaluation measures. This test assumes there is no significant differences between the results of the control method (i.e., SMAOBL) and other compared methods. This assumption is named null hypothesis, and it is accepted if the value of p-value is greater than 0.05. Otherwise (i.e., p-value less than 0.05), it was rejected, and this confirms that the difference between SMAOBL and other methods is significant.

As indicated in Table 6, the proposed SMAOLB recorded the best Friedman’s value in terms of RSME, MAE, and MAPE. The GA obtained the second rank for both MAE and MAPE, followed by PSO, SMA, GWO, ANFIS, and SCA. For RMSE, the PSO obtained the second rank, followed by GA, SMA, GWO, ANFIS, and SCA.

**Table 6:** The results of the Friedman test

	ANFIS	SMA	SMAOBL	PSO	GA	SCA	GWO
MAE	5.462	3.769	**2.077**	2.462	2.231	6.923	5.077
RMSE	5.385	4.000	**1.462**	2.462	2.615	7.000	5.077
MAPE	5.462	3.923	**2.000**	2.385	2.231	7.000	5.000

In summary, the above-mentioned results ensured the competitive performance of the developed SMAOLB-ANFIS over the traditional ANFIS and the modified ANFIS using SMA. More so, it outperformed several optimizers that are applied to improve the ANFIS model, such as PSO, GA, SCA, and GWO. Since the developed SMAOBL combined the strength of the SMA and the OBL strategy that aims to support SMA with a suitable mechanism to avoid stuck in local optima, this has been performed during the exploration phase, and this leads to increase the convergence rates towards the feasible regions which contain the optimal solutions (parameters of ANFIS).

## Conclusion

This study proposed a developed variant of the ANFIS model, as a time-series forecasting method for oil production using real-world datasets. The traditional ANFIS was enhanced using an intelligence optimization method called SMAOLB. This method was developed by applying the intelligence OLB technique to improve the search process of the slime mould algorithm (SMA). Thus, the proposed forecasting model called ANFIS-SMAOLB was applied to forecast oil production using different datasets from two real-world oilfields in Yemen and China. We implemented several experiments considering several evaluation metrics and statistical tests to evaluate the performance of the developed ANFIS-SMAOLB. Additionally, we compared it to the original structure of the ANFIS and several modified ANFIS using other optimization mechanisms, such as traditional SMA, SCA, PSO, GA, and GWO. We concluded that the SMAOLB showed better performance than the traditional ANFIS, SMA, and other ANFIS versions in all performance measures, except the computational time (CPU time). Therefore, the main limitation of the developed SMAOLB is the computational time, which can be neglected compared to other performance measures that have more important roles in time series prediction and forecasting, such as $R^2$, RMSE, MAE, MAPE, and STD. For future work, there are other applications that could be addressed using the SMAOLB, such as feature selection, multi-optimization tasks, and scheduling tasks (i.e., cloud computing, machine job scheduling in manufacturers).
